# SNP rs11185644 of RXRA gene is identified for dose-response variability to vitamin D3 supplementation: a randomized clinical trial

**DOI:** 10.1038/srep40593

**Published:** 2017-01-12

**Authors:** Mingzhi Zhang, Lan-Juan Zhao, Yu Zhou, Rhamee Badr, Patrice Watson, An Ye, Boting Zhou, Jigang Zhang, Hong-Wen Deng, Robert R. Recker, Joan M. Lappe

**Affiliations:** 1 School of Public Health, Medical College of Soochow University, Department of Epidemiology & Biostatistics, Suzhou, 215123, China; 2 Center for Bioinformatics and Genomics, Department of Biostatistics and Bioinformatics, Tulane University, New Orleans, LA, 70112, USA; 3 Jiangsu Key Laboratory of Preventive and Translational Medicine for Geriatric Diseases, Medical College of Soochow University, Suzhou, 215123, China; 4 School of Medicine, Tulane University, New Orleans, LA, 70112, USA; 5 Osteoporosis Research Center, Creighton University, Omaha, NE, 68131, USA

## Abstract

The level of serum 25-Hydroxyvitamin D [25(OH)D] has high heritability, suggesting that genes may contribute to variations in serum 25(OH)D level and vitamin D dose-response. As vitamin D deficiency has been linked to numerous diseases, understanding how genetic variation contributes to vitamin D dose-response is important for personalized vitamin D treatment and cost-effective disease prevention. To identify genetic variants responsible for vitamin D status and dose-response, we performed two vitamin D3 and calcium clinical supplementation trials in 2,207 postmenopausal Caucasian women. We examined the association of 291 SNPs with baseline serum 25(OH)D levels and 25(OH)D dose-response. Five SNPs, rs10500804 (*P* = 4.93 × 10^−7^), rs2060793 (*P* = 6.63 × 10^−7^), rs10741657 (*P* = 1.49 × 10^−6^), rs10766197 (*P* = 1.05 × 10^−5^) and rs11023380 (*P* = 7.67 × 10^−5^) in the CYP2R1 gene, as well as 6 SNPs, rs4588 (*P* = 7.86 × 10^−7^), rs2298850 (*P* = 1.94 × 10^−6^), rs1155563 (*P* = 6.39 × 10^−6^), rs705119 (*P* = 2.80 × 10^−5^), rs705120 (*P* = 1.08 × 10^−4^) and rs222040 (*P* = 1.59 × 10^−4^) in the GC gene were associated with baseline serum 25(OH)D levels. SNP rs11185644 near the RXRA was significantly associated with 25(OH)D dose-response (*P* = 1.01 × 10^−4^). Our data suggest that polymorphisms in the CYP2R1 and GC gene may contribute to variation in baseline serum 25(OH)D concentration, and that polymorphism rs11185644 may contribute to variation in 25(OH)D dose-response in healthy postmenopausal Caucasian women.

Vitamin D deficiency is a health issue that may particularly affect the elderly population. Serum 25-hydroxyvitamin D (25(OH)D) is the best biomarker for assessing vitamin D deficiency or insufficiency^1^. The optimal vitamin D status was defined as serum 25(OH)D concentrations exceeding 75 nmol/L by the Endocrine Society^2^. Low levels of serum 25(OH)D are associated with many chronic conditions, such as osteoporosis^3^,^4^, diabetes^5^,^6^, cardiovascular diseases^7^,^8^,^9^,^10^, cancer^11^,^12^, depression^13^, lupus^14^, and chronic kidney disease^15^,^16^. Based on results from our previous population-based study of vitamin D status in postmenopausal women in Eastern Nebraska, we extrapolate that more than 60% of elderly female North Americans may have serum 25(OH)D concentrations below 75 nmol/L^17^.

Vitamin D, an essential nutrient, is a pro-steroid hormone. Whether obtained through diet, supplement, or produced in the skin, vitamin D is metabolized in the liver to 25-hydroxyvitamin D [25(OH)D] by the enzyme 25-hydroxylase. 25(OH)D is further hydroxylated to 1,25-dihydroxyvitamin D [1,25(OH)_2_D]. The broad function of vitamin D is through 1,25(OH)_2_D, a hormone that regulates ~1000 genes in the human genome^18^,^19^. Optimal local (intracellular) synthesis of 1,25(OH)_2_D is dependent on optimal concentrations of serum 25(OH)D. Therefore, achieving and maintaining an optimal serum 25(OH)D level is essential for tissues to efficiently synthesize hormone 1,25(OH)_2_D for overall body health^20^.

Currently, vitamin D supplementation is the best way to achieve adequate serum vitamin D levels^21^. There are two types of vitamin D supplements available: vitamin D2 and D3. Vitamin D3 supplementation is widely recommended by clinicians for the prevention and treatment of bone disease. Vitamin D along with Calcium supplementation has been shown to decrease fracture risk in postmenopausal women with osteoporosis as well as older men^22^,^23^. Studies have also found potential benefits associated with vitamin D in the management of a multitude of conditions, such as statin-induced myalgia and type 2 diabetes^24^,^25^. However, the change in serum vitamin D in response to a given dose of vitamin D supplementation varies widely among individuals. Body size, baseline serum 25(OH)D level, season in which vitamin D supplementation is initiated, as well as baseline serum Calcium levels^26^,^27^,^28^,^29^,^30^,^31^ have all been associated with variation in the increase in serum 25(OH)D following vitamin D supplementation.

Genetics may also play an integral role in response to vitamin D intake. Genome wide association studies^32^,^33^ have demonstrated that serum vitamin D levels are influenced by genotype, however, few studies have examined the impact of genetic variation in response to vitamin D supplementation. The sparse data that do exist on this matter^26^,^34^,^35^,^36^ have implicated SNPs in DBP/GC (vitamin D binding protein) and CYP2R1 (cytochrome P450, family 2, subfamily R, polypeptide 1) are associated with vitamin D dose response. To the best of our knowledge, no other genes have yet been found to modify the response to vitamin D supplementation. Identification of the genetic variants responsible for the wide variation in vitamin D status and vitamin D dose-response is important for development of personalized vitamin D treatment plans and cost-effective disease prevention protocols in osteoporosis and other diseases^37^,^38^,^39^,^40^. Based on data from two completed vitamin D clinical trials, we examined the effect of polymorphisms in 15 candidate genes on vitamin D dose-response variation in postmenopausal women. The 15 genes included in our study were carefully selected based on their functional importance for vitamin D metabolism, transportation, and signaling pathways. The association of these candidate genes to baseline serum 25(OH)D was also assessed.

## Results

### General characteristics of 2,207 subjects in the CaMEWS and D&Cancer study

A total of 2,207 subjects with complete data were included in the genetic association analysis of baseline serum 25(OH)D variation. Across both cohorts, the average age of participants was 65.1 years, mean BMI was 29.8, and mean serum 25(OH)D level at baseline was 79.4 nmol/L. The range of baseline serum 25(OH)D level ranged from 24.2 to 144.6 nmol/L in the CaMEWS, and from 13.3 to 225.8 nmol/L in the D&Cancer study.

General characteristics of the cohorts by group are presented in Table 1. In both study cohorts, age, height, weight, BMI, baseline serum 25(OH)D and baseline serum calcium were not significantly different between the control and intervention groups (all *P* > 0.05). As expected, the calcium and vitamin D groups had significantly greater changes in serum 25(OH)D levels than either the calcium only group or the placebo group (*P* < 0.0001). For Calcium + Vitamin D treatment groups, our measurements showed a mean serum 25(OH)D change of +24.31 and +31.92 nmol/L in the CaMEWS and D&Cancer studies respectively compared to mean serum 25(OH)D changes of −1.02 nmol/L for the Calcium only group of the CaMEWS study and 0.36 nmol/L for the D&Cancer study. There was no difference in serum calcium change between treatment and control groups.

### Genes responsible for vitamin D dose-response variation

In the gene association analysis of serum 25(OH)D response, only one SNP, rs11185644, was identified to be significantly associated with serum 25(OH)D response. SNP rs11185644 was near the RXRA (Retinoid X receptor, alpha) gene. The Wald test unadjusted *P* value was 1.01 × 10^−4^. After Bonferroni adjustment, this association remained significant (*P* = 0.029). The two alleles of the SNP are A and G, the MAF was equal to 0.17, and the *P* value for HWE test was 0.006.

Serum 25(OH)D changes and serum 25(OH)D response according to genotypes of rs11185644 were presented in Table 2. Doses of vitamin D stratified by genotype were also presented. After adjusting for age, BMI, and phlebotomy season, serum 25(OH)D response was significantly different among genotypes (*P* < 0.001). Subjects with genotype GG had higher serum 25(OH)D responses compared with those with genotype AA or AG carriers.

### Genetic variants responsible for baseline serum 25(OH)D levels

With age, phlebotomy season and BMI adjusted, eleven SNPs were identified to be significantly associated with baseline serum 25(OH)D in the overall population and in the subgroups. Characteristics of the 11 SNPs and their association with baseline serum 25(OH)D were listed in Table 3. Four SNPs in the CYP2R1 gene were found to be associated with baseline serum 25(OH)D in the D &Cancer study. Three of these SNPs, rs2060793, rs10741657, and rs10766197, are located in the gene promoter and the SNP rs10500804 was found in an intron segment. Six SNPs in the GC gene were found to be associated with baseline serum 25(OH)D in the CaMEWS study: Intron SNPs rs2298850, rs1155563, rs705119, rs705120 and rs222040, and missense SNP rs4588. Besides their significance in subgroups, these 10 SNPs and another SNP rs11023380 (in the promoter of CYP2R1) identified were also significantly associated with baseline serum 25(OH)D in the overall population (n = 2207) (*P* < 2.0 × 10^−4^) (Table 3). The results remain significant even after Bonferroni correction (*P* < 0.05).

## Discussion

Our study examined the association of fifteen candidate genes with baseline serum 25(OH)D and serum 25(OH)D responses to vitamin D supplementation in postmenopausal Caucasian women. Our data suggest that CYP2R1 and GC gene polymorphisms are associated with baseline serum 25(OH)D and that SNP rs11185644 polymorphisms have a statistically significant association with vitamin D dose response. These findings confirm what other studies have demonstrated with regards to the effects of GC and CYP2R1 on baseline serum 25(OH)D; however, we were not able to replicate any association between these genes and vitamin D dose response. Additionally, our study is the first to implicate the potential effect of SNP rs11185644 on vitamin D dose response.

CYP2R1, a member of the CYP2 family, encodes cytochrome p4502R1. Previous studies have shown that the CYP2R1 gene is associated with several vitamin D related diseases including type 1 diabetes^41^, polycystic ovary syndrome^42^, and ovarian cancer^43^. As a microsomal vitamin D 25-hydroxylase in humans, cytochrome P450 2R1 converts vitamin D into 25(OH)D. Given that it is responsible for 25(OH)D productions from a biological precursor, it is very plausible for mutations in this gene to contribute to variations in baseline serum 25(OH)D, and indeed this has been shown to be the case. An inherited transition mutation in exon 2 of CYP2R1 has been demonstrated to eliminate its 25-hydroxylase activity and was associated with low circulating levels of 25(OH)D and classic symptoms of vitamin D deficiency^44^. SNPs in CYP2R1 were previously reported to be associated with serum vitamin D levels in two large genome - wide association studies (GWAS)^32^,^33^ in Caucasian cohorts as well as several candidate gene studies, including one of 1204 postmenopausal women of European descent^45^. Our study demonstrates a statistically significant association of serum 25(OH)D with five SNPs in the CYP2R1 gene (rs2060793, rs10500804, rs11023380, rs10741657, rs10766197) and each of these has been replicated by other studies. Specifically, rs2060793, rs10500804 and rs11023380 were replicated by Engleman’s candidate gene study in a summer phlebotomy group^45^; rs2060793 was also identified in Ahn’s GWAS^32^ of 4,501 persons of European ancestry (*P* = 2.9 × 10^−17^); rs10741657 was included in Wang’s GWAS^33^ of 30,000 individuals of European descent (*P* = 3.3 × 10^−20^). Lastly, rs10766197’s association with serum 25(OH)D levels was demonstrated in healthy Danish children and adults^46^.

GC, the vitamin D binding protein gene, encodes DBP, a 52–59 kDA protein synthesized in the liver that binds and transports vitamin D and its metabolites (including 25(OH)D and 1,25(OH)_2_ D)^47^. The portion of 25(OH)D bound to vitamin D binding protein makes up by far the largest share of the total serum 25(OH)D (the other smaller portions represent the albumin bound and free fraction)^48^; hence there is certainly biological plausibility for a GC polymorphism contribution to variability in total serum 25(OH)D. Multiple studies have reported that several nonsynonymous SNPs in this gene are associated with 25(OH)D concentrations^45^,^49^,^50^,^51^. Nonsynonymous SNPs rs4588 (Thr → Lys) and rs7041 (Asp → Glu) are the most common *GC* variants^45^,^51^. In Engelman’s study^45^, rs4588 was significantly associated with 25(OH)D with an uncorrected *P* value of less than 0.001 (Bonferroni-corrected significance of a/n of SNPs tested = 0.05/29 = 0.0017). Our data strongly replicates this association (Bonferroni single-step adjusted *P*-value = 2.08 × 10^−4^). Another GC polymorphism frequently associated with serum 25(OH)D is rs7041. Wang’s GWAS found a strong association of rs7041 with circulating 25(OH)D (overall meta-analytic *P* = 1.9 × 10^−109^), Engleman demonstrated an initial P value for rs7041′s association with serum 25(OH)D of 0.02, however after association analysis of haplotype blocks it was determined that rs7041 was not independently significant. We were unable to successfully genotype rs7041 in our study. Given conflicting evidence, more research may be necessary to further elucidate the relationship between GC polymorphism, rs7041, and serum 25(OH)D levels. What remains clear is that polymorphisms in both CYP2R1 and GC may have considerable phenotypic effects on serum 25(OH)D concentrations. The clinical relevance of these polymorphisms and their prevalence remain unknown.

In a previous study conducted by our group of investigators, we demonstrated that increase in vitamin D intake, baseline serum 25(OH)D level, baseline blood collection season, baseline serum calcium level, and baseline BMI were associated with serum 25(OH)D response variation^52^, a finding consistent with the current body of literature. Taken together, these five factors account for 46.8 percent of the vitamin D response variation, however this still leaves a significant portion of the total variation unaccounted for. Several recent studies have suggested that genetics plays a role in response to vitamin D intake^26^,^34^,^35^,^36^, potentially bridging this gap. Two genes thus far implicated in this process are CYP2R1 and GC, both of which have already been shown (both within this paper and by others) to be involved in regulating baseline serum 25(OH)D. Fu *et al*. showed that the rs4588 SNP in GC was associated with response to vitamin D supplementation with the highest increase in serum 25(OH)D levels seen with the minor homozygote genotype. Nimitphong, in a small Thai study, replicated these findings^34^. Waterhouse *et al*. in a much larger study, did not find any statistically significant association of rs4588 and response to vitamin D supplementation^35^. Our study echoed these later findings, as we could not establish any significant association between rs4588 and vitamin D dose response (*P* = 0.1523). The 2009 study by Fu *et al*. included only 98 adults and the 2013 study by Nimitphong *et al*. had only 41 subjects, all of them of Thai decent. Given the superior sample size, breadth, and statistical power of both our study and the Waterhouse study (358 and 385 participants with genetic analysis as well as measured baseline and follow up serum 25 (OH)D values respectively) relative to the Fu *et al*. and Nimitphong *et al*. studies, we feel that GC, and specifically rs4588, is not significantly associated with vitamin D response variation. However, CYP2R1 gene may be involved. Waterhouse *et al*. found that SNP rs10766197 in CYP2R1 was associated with vitamin D dose response, with the greatest increase in serum 25(OH)D concentration reported for those homozygous for the major allele (they also demonstrated an association of this SNP and baseline 25(OH)D levels as we have). Didrikson *et al*. came to the same conclusion in their study. Our data failed to demonstrate any statistically significant association for CYP2R1 SNP rs10766197 and vitamin D dose response (*P* = 0.20). Given these divergent results, further research may be necessary to reach a consensus on CYP2R1 and its regulation of vitamin D dose response.

Although our study demonstrated no statistically significant association of CYP2R1 and GC polymorphisms with vitamin D dose response, we were able to illustrate the association of SNP rs11185644 with serum 25(OH)D response to vitamin D supplementation after adjusting for age, BMI, and phlebotomy season (*P* = 1.01 × 10^−4^). This polymorphism, which has barely to be described in the literature thus far, lies in upstream of the transcription start sites of the RXRA gene. The RXRA gene encodes the retinoic X receptor alpha, which is one of the nuclear receptors that mediate the biological effects of retinoid via their involvement in retinoic acid-mediated gene activation. So far, the longest distance scanned for RXRA promoter is 5 kb upstream of the translation start site (TSS)^53^. Therefore, the SNP may not belong to the promoter region of the RXRA. The SNP is located in intergenic region, between gene RXRA and the uncharacterized LOC105376310. It is ~7.5 kb near the 5′ of the RXRA gene and ~24 kb ahead of the 3′ of the LOC105376310. The identified significant polymorphism, SNP rs11185644, is barely studied or reported. Only one paper which investigated the relationship between this SNP and pancreatic adenocarcinoma^54^. This is the first study reporting the association between SNP rs11185644 and vitamin D dose response. Based on limited information of the SNP, its function is largely unknown.

Previous research has demonstrated that differences in serum 25(OH)D levels between the genotypes studied were most pronounced after 6 months of vitamin D supplementation, implying that mechanisms underlying CYP2R1’s and GC’s effects on response to vitamin D supplementation may be time-sensitive and may perhaps reach a saturation point^26^. Didrikson’s findings suggest that this study’s results and associations may have differed if the time scale for follow-up measurements was shortened.

This study measured the total serum 25(OH)D levels; however, there is the possibility that the free 25(OH)D level is related to some phenotypes associated with variations in vitamin D levels, such as bone mineral density^55^, and that the free 25(OH)D levels may be subject to genetic influences as well. Future research should measure the biologically active free 25(OH)D levels in relation to SNPs in CYP2R1, GC, and other implicated genes.

Our findings are noteworthy because they are based on a large, well control clinical trial study. The power estimation under current parameter setting is over 95%. Our homogeneous study population also contributed to the reliability of our findings. All subjects were postmenopausal Caucasian women from the same geographical area in rural Nebraska (41.4 degrees North latitude). Adherence to vitamin D supplementation was also considered, adding to the validity of our data. Furthermore, using 12-month vitamin D supplementation data reduced the confounding effect of season. There are, however, a few limitations to the current study. First, Due to the nature of the clinical trials, the parent studies did not consider data regarding dietary vitamin D intake and sun exposure. Information about medication or additional pathologies are not included in the exclusion criteria, which may affect vitamin D status. For instance, GI pathology or medications affecting gut absorption of vitamin D may influence the results. Although such information was not collected, the randomization that we did for the two clinic trials should reduce the confounding effect of these factors. Second, in drafting a list of genes and polymorphisms for association analysis, a gene responsible for cholecalciferol production in the skin^45^, DHCR7/NADSYN, was not included in our study. A Swiss study recently implicated this gene in vitamin D metabolism, finding that SNPs in DHCR7 were associated with serum 25(OH)D levels in northeastern Han Chinese children^56^. Third, the doses of vitamin D intervention were different in the two studies, 1100 IU/day for CaMEWS and 2000 IU/day for D&Cancer. Although vitamin D3 with 1100 IU/d or 2000 IU/d was administered, not all subjects followed the instruction and took the required pills each day. In the study, adherence rate was evaluated. The doses of vitamin D of the interventions were adjusted and standardized. Serum 25(OH)D dose-response was served as the phenotype. Therefore, we don’t think the different vitamin D3 doses were a big problem for the study. Lastly, this research was limited to postmenopausal Caucasian women, therefore limiting the generalizability of this study’s findings. However, these preliminary findings present an opportunity for further research in different population groups; African Americans, for instance, have lower baseline vitamin D levels than their white counterparts^57^,^58^, and should be considered for inclusion in future research. Our study demonstrates that genetic polymorphisms in genes involved with vitamin D metabolism and function may impact serum vitamin D levels in one particular cohort. Further research is required to elucidate whether these polymorphisms and others yet discovered partially underlie racial differences in vitamin D.

## Methods

### Subjects and trial design

The subjects for this study are non-Hispanic white postmenopausal women from two previously completed vitamin D3 intervention studies. Both studies were population-based, randomized, placebo-controlled, double blinded, calcium and vitamin D3 intervention clinical trials. Samples from the two parent studies were demographically similar. The participants were randomly selected from a rural area of Nebraska (approximately 41.4 degrees N) and were all ≥ 55 years.

The subject inclusion criteria for the two parent studies were: (1) good general health; (2) ≥ 4 years post menopause; and (3) the ability to live independently and travel to the Creighton University Osteoporosis Research Center (ORC) Study Site at Fremont Area Medical Center (FAMC). Subjects were excluded if they exhibited: (1) any history of cancer or other malignancies treated curatively < 10 years ago, except superficial basal or squamous cell carcinoma of the skin; (2) known metabolic bone diseases; (3) chronic kidney disease; (4) Paget’s disease; (5) co-morbidity of tuberculosis or sarcoidosis. All subjects provided written informed consent for the study. The Institutional Review Board at Creighton University (Omaha, NE) approved both studies. Safety was assessed in participants who received Calcium and vitamin D with the given dose.

The first parent study “Calcium and Vitamin D Malnutrition in Elderly Women Study” (CaMEWS) began in 2000 and was completed in 2005. The participants have been described in detail in our other papers^17^,^59^. Briefly, 1,179 non-Hispanic white postmenopausal women were enrolled, and assigned to receive either 1500 mg/d supplemental calcium alone (Calcium only group), supplemental calcium (1500 mg/d) plus 1100IU/d vitamin D3 (Ca + D group), or double placebos (placebo group). Supplements were given to the subjects by the project nurses at each 6-mo visit.

The second parent study, “Clinical Trial of Vitamin D3 to Reduce Cancer Risk in Postmenopausal Women (D&Cancer),” was a population–based sample of 2,300 healthy postmenopausal women from 2009 and was completed in 2015. Half of the sample (n = 1,150 subjects) received supplements of vitamin D3 (2000 IU/day) and calcium (1500 mg/day) (Ca + D group); the remaining half received placebos of vitamin D3 and calcium (placebo group).

For both studies, vitamin D3 was made by Tishcon Corp (Westbury, NY), supplied such that each capsule had 2000 IU for D&Cancer study and 1100 IU for CaMEWS. Each subject received one capsule of vitamin D or identical placebo based on their assigned group. Subjects can’t tell the difference of the capsule of vitamin D or placebo from their appearance. To ensure the quality of the drug, we used each batch of capsules within one year. We analyzed a sample of each lot of vitamin D3 when we received it and at the end of each year to assure potency.

In the double blinded clinical trials, participants were randomly assigned to the groups before their baseline circulating vitamin D levels were measured. Because of the high incidence of osteoporosis in the postmenopausal women and because some subjects would be given placebos, we believe it would be unethical to advise women to avoid self- supplementation. Participants would be informed that they may be assigned to placebo calcium and vitamin D. Participants were allowed to take their own vitamin D, but we asked them to limit that to no more than 400 IU/day if they are < 70 years of age and to no more than 600 IU/day if they are ≥ 70, which are the currently recommended intake levels.

The two trials were both registered at ClinicalTrials.gov website (NCT00352170 for CaMEWS study and NCT01052051 for D&Cancer study) on 12 July 2006 and 19 January 2010 respectively, and ClinicalTrials.gov processed this record on April 05, 2016. No changes to the methodology occurred following trial commencement. We confirmed that all methods were performed in accordance with the approved guidelines and regulations. We report and present data according to the CONSORT statement.

In order to identify genes for dose-response variability to vitamin D3 supplementation, we need subjects who have DNA available. The original clinical trials did not intend to collect whole blood and have DNA extracted. With the limited fund, after the completion of the CaMEWS (which were completed in 2005), of the 1,179 subjects, we reenrolled 358 women for their blood draw and DNA extraction. Of the 358 subjects, 155 participants were in the calcium-only group while 203 participants were in the Calcium + Vitamin D group (Table 1). For the D&Cancer, of the 2,300 participants, with the limited fund, 1,849 women had their DNA samples extracted and have vitamin D response data for the study. Of the 1,849 subjects, 934 of these measurements came from the placebo group, while 915 came from the Calcium + Vitamin D group (Table 1).

In summary, a total of 2,207 subjects (358 subjects from CaMEWS and 1,849 subjects from D&Cancer study) were included in the analysis of gene association with baseline serum 25(OH)D levels. Table 1 lists the characteristics of the 2,207 subjects used for this study.

### Primary and secondary outcome measures

The primary outcome measure of the first parent study (CaMEWS study) is fractures, and the secondary outcome measures are changes in bone mass and density, changes in serum 25(OH)D measurement and so on. The primary outcome measure of the second parent study (D&Cancer) is a cancer diagnosis, and the secondary outcome measures are some chronic diseases. No change occurred to trial outcomes after the trials commenced. In the current study, the primary outcome measure is serum 25(OH)D dose-response variation. The dose-response variation was computed as: Serum 25(OH)D dose-response = (Serum 25(OH)D level after 12-month intervention- Serum 25(OH)D level at baseline)/Total vitamin D supplement intake). The secondary outcome measure is baseline serum 25(OH)D.

### Clinical measurements

For both CaMEWS and D&Cancer, blood was drawn at baseline and again after 12-month intervention. Blood was collected after a 3-hour fast, and participants were asked to not take vitamin or mineral supplements the morning of the phlebotomy. The blood collection dates were transformed into three periods (December through February, the first period with poor UVB exposure from sunlight; March through May, and September through November, the second period with plentiful UVB exposure from sunlight; June through August, the third period with strong UVB exposure from sunlight).

Serum 25(OH)D concentration was measured by RIA (Nichols/Quest Diagnostics, San Clemente, CA or DiaSorin assay (Stillwater, MN) for each participant at baseline and after 12-month intervention. The measurement combines 25-hydroxyvitamin D2 and 25-hydroxyvitamin D3, thus, only total 25-hydroxyvitamin concentration were reported. The measurement can’t quantitates 25(OH)D2 and 25(OH)D3 separately. All biochemical analyses were completed in a single laboratory that participates in the Quality Assurance Program for vitamin D. Other important variables that may influence variation in serum 25(OH)D, such as age, weight, height, total calcium supplement, serum calcium levels, blood collection dates, adherence to the trial vitamin D supplementation, and self-selected vitamin D supplementation were collected at baseline and at the end of study. BMI (body mass index) was defined as weight (kilograms) divided by height squared (square meters). Compliance with trial vitamin D supplementation was assessed at 6-month intervals by bottle weight and mean adherence (defined as taking 80% of assigned doses) was 86%^17^.

The phenotypes for the study were baseline serum 25(OH)D variation and serum 25(OH)D dose-response variation.

In both clinical trials, the trial vitamin D supplement was calculated as per-protocol supplement dose adjusted for individual compliance rates. Adherence with supplementation were determined by weighting each bottle of vitamin D3 tablets before distributing the bottle and after it was returned by the participant every 6 months. We initially determined the weight of each pill: calcium, vitamin D3, placebo calcium and placebo D3. To do this, we weighed the empty bottle and then the full bottle. Then we subtracted the weight of the empty bottle from the weight of the full bottle and divided the difference by the number of tablets in the bottle. Participants do not evaluate their compliance. We assess the compliance at six-monthly intervals by bottle weight. Bottles were dark so that light could not penetrate.

In addition to the administrated trial vitamin D, subjects were allowed to take their self-selected vitamin D supplement. The self-selected vitamin D supplementation was measured at baseline and at the end of the study. The average amount of supplement was calculated. The total vitamin D supplement intake, including the self-selected vitamin D supplement and the trial vitamin D supplement, was used for analysis.

### Candidate genes

Fifteen candidate genes were selected according to the following criteria: (1) evidence of significant association in previous studies; (2) biological importance in vitamin D metabolism, transportation, degradation, or vitamin D signaling pathways. The fifteen genes were CYP24A1 (vitamin D3 24-hydroxylase), CYP27A1 (vitamin D3 25-hydroxylase), CYP27B1 (25 - OH D-1-alpha hydroxylase), CYP2R1, RXRA (Retinoid X receptor, alpha), RXRB(Retinoid X receptor, beta), CYP3A4 (cytochrome P450, family 3, subfamily A, polypeptide 4), GC, VDR (vitamin D receptor), PTH (parathyroid hormone), CYP11A1 (Cytochrome P450, family 11, subfamily A, polypeptide 1). CYP1A1 (Cytochrome P450, family 1, subfamily A, polypeptide 1), CASR (Calcium-sensing receptor), TNF (Tumor necrosis receptor), and FGF23 (Fibroblast growth factor 23). The general characteristics of the fifteen genes are included in Table 4.

### SNP genotyping

We initially selected 348 SNPs within and around our 15 candidate genes. Tag SNPs, which have minor allele frequency (MAF) > 5% in the HapMap CEU population, were selected via the software program SNPbrowser (v4.0.1). The tag SNP selection is based on the HapMap database (release 20, January 24, 2006) with two methods: pair-wise *r*^2^ (*r*^2^ ≥ 0.8) and haplotype *R*^2^ (*R*^2^ ≥ 0.8)^60^. In addition to tag SNPs, we chose other SNPs in the promoter, including 3′UTR, and exon regions that indicate potential functional importance.

For each subject, genomic DNA was extracted from peripheral blood using the Puregene DNA isolation kit (Qiagen Inc.) following the provided protocol. DNA samples were diluted to 20 · g/ml and shipped to iGenix Inc (Bainbridge Island, WA) for SNP genotyping using the Illumina GoldenGate Genotyping Assay. Based on the genotyping results, SNPs with low call rate (<5%) were discarded. SNPs departed from Hardy-Weinberg equilibrium (HWE) at the p < 0.00001 were removed from data analyses. In total, 291 SNPs were selected for genotyping analysis. All chosen SNPs were confirmed from NCBI (http://www.ncbi.nlm.nih.gov/SNP/) and HapMap (http://www.hapmap.org).

### Interim analyses and stopping guidelines

Not applicable.

### Randomization

The study statistician generated the randomization sequence with the use of a computer-generated permuted blocks (*n* = 5) randomization scheme, and the study nurses enrolled the subjects and assigned them to groups. By design, the 2 active treatment groups were each allocated ≈40% of the cohort, and the placebo group 20%. Participants and the study nurses were both blinded.

### Statistical data analyses

For the baseline serum 25(OH)D variation analysis, 2,207 subjects were included. Characteristics of the two study cohorts were described in Table 1 with mean and standard deviation figures included for both trial and control groups and compared using the t test. One-sample Kolmogorov-Smirnov tests were conducted for testing the normal distribution for each descriptive variable. For the association analysis of serum baseline 25(OH) variation and serum 25(OH)D dose-response variation with the candidate genes, the phenotype was adjusted using a linear regression model as age, BMI and phlebotomy season (December-February; March-May and September-November; June-August) considered as potential covariates. All the statistical analyses were performed with SAS version 9.2(SAS Institute Inc., Cary, North Carolina). All t tests were done two tailed, and *P* < 0.05 was considered statistically significant. *P* < 0.10 was considered statistically significant for Kolmogorov-Smirnov tests and multiple linear regression analyses.

For the analysis of gene association with serum 25(OH)D response, the 662 women with complete data of 1,118 women who were assigned to the intervention group to receive supplemental vitamin D3 and calcium, were used for the data analysis.

For genetic association analyses, the Hardy-Weinberg equilibrium (HWE) of the genotypic frequencies was examined for subjects in the two cohorts with significance level of 0.00001. A total of 265 SNPs with MAF > 0.05 and P > 0.00001 for Hardy-Weinberg equilibrium testing were included in the data analyses. The candidate gene association test was conducted with PLINK software^61^. Residuals of multiple linear regression analyses for serum baseline 25(OH)D and serum 25(OH)D dose-response adjusted by age, BMI, and phlebotomy season were used as the phenotypes for genetic association analyses. Wald test unadjusted *P*-values, Bonferroni single-step adjusted *P*-values (BONF-*P*), regression coefficients and squared correlation coefficients were calculated. The nominal significance level was set as 0.05 for BONF-*P*.

## Additional Information

**How to cite this article**: Zhang, M. *et al*. SNP rs11185644 of RXRA gene is identified for dose-response variability to vitamin D3 supplementation: a randomized clinical trial. *Sci. Rep.* **7**, 40593; doi: 10.1038/srep40593 (2017).

**Publisher's note:** Springer Nature remains neutral with regard to jurisdictional claims in published maps and institutional affiliations.

## Supplementary Material

Supplementary Information

## Figures and Tables

**Table 1:** Characteristics of the study sample of 2,207 non-Hispanic white postmenopausal women (mean ± SD).

	CaMEWS			D&Cancer study		
	Calcium only (n = 155)	Calcium + Vitamin D (1100 IU/day) (n = 203)	*P* value	Placebo (n = 934)	Calcium + Vitamin D (2000 IU/day) (n = 915)	*P* value
Age (yr)	64.9 ± 6.0	65.2 ± 7.1	0.69	65.3 ± 7.1	65.0 ± 6.9	0.34
Height(cm)	163.5 ± 6.1	162.4 ± 6.3	0.11	162.0 ± 6.3	162.0 ± 6.2	0.78
Weight(kg)	77.4 ± 13.9	75.8 ± 13.3	0.27	79.6 ± 17.8	79.2 ± 18.4	0.68
Body mass index (kg/m^2^)	29.0 ± 5.1	28.7 ± 4.8	0.62	30.3 ± 6.4	30.2 ± 6.8	0.63
Baseline Serum 25(OH)D (nmol/L)	73.4 ± 21.6	74.1 ± 18.5	0.77	80.8 ± 31.7	80.1 ± 25.5	0.57
Baseline Serum calcium (mg/dL)	9.3 ± 0.4	9.3 ± 0.5	0.88	9.4 ± 0.4	9.4 ± 0.3	0.97
Serum 25(OH)D change (nmol/L)	−1.02 ± 11.12	24.31 ± 17.02	**<0.0001**	0.36 ± 40.17	31.92 ± 43.96	**<0.0001**
Serum calcium change (mg/dL)	0.19 ± 0.45	0.23 ± 0.15	0.42	−0.05 ± 0.31	−0.01 ± 0.35	**0.016**

Note: CaMEWS: Calcium and Vitamin D Malnutrition in Elderly Women Study. D&Cancer: Clinical Trial of Vitamin D3 to Reduce Cancer Risk in Postmenopausal Women. *P* value: The *P* value is for difference between two groups in each cohort.

**Table 2:** Serum 25-OH D change and serum 25-OH D response to one-year vitamin D3 intake according to genotype of rs11185644 near RXRA gene: Mean (95% CI)^a^.

Genotype	Dose of vitamin D^b^ (IU)	Serum one-year 25-OH D change (nmol/L)	Serum 25-OH D response (nmol/L/IU) c
D&Cancer (n = 470)			
A A(n = 325)	1782.85 ± 353.31	30.27(26.64, 33.90)	0.016(0.003, 0.029) *
A G(n = 136)	1835.71 ± 294.98	35.61(30.48, 40.74)	0.023(0.006, 0.042) *
G G(n = 9)	1683.25 ± 653.74	36.18(18.09, 54.26)	0.250(0.187, 0.314)
*P* value for difference of genotype	0.201	0.150	**<0.0001**
CaMEWS (n = 192)			
A A(n = 137)	1263.76 ± 351.30	23.69(20.49, 26.90)	0.020(0.017, 0.023)
A G(n = 51)	1216.61 ± 287.92	26.13(21.29, 30.97)	0.021(0.016, 0.026)
G G(n = 4)	1294.73 ± 517.89	28.76(11.77, 45.75)	0.023(0.006, 0.041)
*P* value for difference of genotype	0.674	0.605	0.817
Overall (n = 662)			
A A(n = 462)	1631.78 ± 424.10	28.56(25.88, 31.25)	0.017(0.008, 0.026) *
A G(n = 187)	1663.55 ± 403.45	33.26(29.36, 37.17)	0.022(0.010, 0.036) *
G G(n = 13)	1563.70 ± 621.93	35.33 (21.51, 49.15)	0.182(0.136, 0.227)
*P* value for difference of genotype	0.556	0.076	**<0.0001**

Note: ^c^Vitamin D response = serum 25-OH D change/dose of total Vitamin D supplement intake.

^a^Age, BMI and phlebotomy season were adjusted.

^b^Total vitamin D supplement intake = the self-selected vitamin D supplementation + the trial vitamin D supplementation, and it was presented with mean ± SD.

^*^There is significant difference compared with the GG genotype, *P* < 0.05.

**Table 3:** Characteristics of significant SNPs associated with baseline serum 25(OH)D via multiple tests among participants.

SNP	Chrome	Gene	Allele	Location(bp)	Function	MAF	HWE	BETA	*R*^2^	Unadj-P*	BONF-*P*#
D&Cancer (n = 1849)											
rs10500804	11	CYP2R1	A/C	14910272	intron	0.44	0.90	−4.93	0.013	1.85 × 10^−6^	4.89 × 10^−4^
rs2060793	11	CYP2R1	A/G	14945310	promoter	0.40	0.37	4.75	0.012	4.49 × 10^−6^	0.001
rs10741657	11	CYP2R1	A/G	14921880	promoter	0.40	0.53	4.64	0.011	9.77 × 10^−6^	0.003
rs10766197	11	CYP2R1	A/G	14914877	promoter	0.47	0.83	−4.24	0.009	3.60 × 10^−5^	0.010
CaMEWS (n = 358)											
rs4588	4	GC	A/C	72618323	missense	0.29	0.64	−9.42	0.096	2.39 × 10^−9^	6.40 × 10^−7^
rs2298850	4	GC	C/G	72614266	intron	0.28	0.75	−8.89	0.087	1.43 × 10^−8^	3.83 × 10^−6^
rs1155563	4	GC	C/T	72643487	intron	0.29	0.80	−8.26	0.081	5.35 × 10^−8^	1.44 × 10^−5^
rs222040	4	GC	C/T	72616931	intron	0.44	0.20	−5.81	0.044	7.48 × 10^−5^	0.020
rs705119	4	GC	A/C	72613035	intron	0.42	0.40	−5.64	0.042	1.07 × 10^−4^	0.029
rs705120	4	GC	G/T	72614139	intron	0.42	0.40	−5.39	0.039	1.80 × 10^−4^	0.048
Overall (n = 2207)											
rs10500804	11	CYP2R1	A/C	14910272	intron	0.44	0.90	−4.51	0.012	4.93 × 10^−7^	1.31 × 10^−4^
rs2060793	11	CYP2R1	A/G	14945310	promoter	0.40	0.37	4.45	0.011	6.63 × 10^−7^	1.76 × 10^−4^
rs4588	4	GC	A/C	72618323	missense	0.29	0.64	−4.86	0.011	7.86 × 10^−7^	2.08 × 10^−4^
rs10741657	11	CYP2R1	A/G	14921880	promoter	0.40	0.53	4.37	0.011	1.49 × 10^−6^	3.94 × 10^−4^
rs2298850	4	GC	C/G	72614266	intron	0.28	0.75	−4.74	0.010	1.94 × 10^−6^	5.15 × 10^−4^
rs1155563	4	GC	C/T	72643487	intron	0.29	0.80	−4.14	0.009	6.39 × 10^−6^	0.002
rs10766197	11	CYP2R1	A/G	14914877	promoter	0.47	0.83	−3.93	0.009	1.05 × 10^−5^	0.003
rs705119	4	GC	A/C	72613035	intron	0.42	0.40	−3.81	0.008	2.80 × 10^−5^	0.007
rs11023380	11	CYP2R1	A/G	14930058	promoter	0.50	0.34	−3.53	0.007	7.67 × 10^−5^	0.020
rs705120	4	GC	G/T	72614139	intron	0.42	0.40	−3.53	0.007	1.08 × 10^−4^	0.029
rs222040	4	GC	C/T	72616931	intron	0.44	0.20	−3.45	0.007	1.59 × 10^−4^	0.042

Note:

^*^Wald test unadjusted *P*-value.

^#^Bonferroni single-step adjusted *P*-value.

**Table 4:** Basic characteristic of the 15 candidate genes.

Acronym	Full name	Region	Length (kb)	Number of exons	Number of Selected SNPs
CYP27A1	Vitamin D (3) 25-hydroxylase	2q35	33.5	9	22
CYP27B1	25(OH)D-1-alpha hydroxylase	2q13.1	4.9	9	4
CYP2R1	Cytochrome P450, family 2, subfamily R, polypeptide 1	11p15.2	14.2	5	12
CYP24A1	Vitamin D (3) 24-hydroxylase	20q13.2	20.5	12	29
GC	Vitamin D binding protein	4q13.3	42.5	13	32
RXRA	Retinoid X receptor, alpha	9q34.3	114.1	10	41
RXRB	Retinoid X receptor, beta	6p21.3	7.1	10	4
VDR	Vitamin D receptor	12q13.1	63.5	11	49
CYP3A4	Cytochrome P450, family 3, subfamily A, polypeptide 4	7q21.1	27.2	13	6
PTH	Parathyroid hormone	11p15.2	4.0	3	7
CYP11A1	Cytochrome P450, family 11, subfamily A, polypeptide 1	15q23	30.0	9	7
CYP1A1	Cytochrome P450, family 1, subfamily A, polypeptide 1	15q24.1	6.0	7	3
CASR	Calcium-sensing receptor	3q13	102.8	6	68
TNF	Tumor necrosis factor	6p21.3	5.2	6	3
FGF23	Fibroblast growth factor 23	12p13.3	25.8	12	4
