# Supplementary material for: SNP rs11185644 of RXRA gene is identified for dose-response variability to vitamin D3 supplementation: a randomized clinical trial
Source: Sci Rep. 2017 Jan 12;7:40593. doi: 10.1038/srep40593 (PMC5228186; doi:10.1038/srep40593)
Supplement: Supplementary Information [file srep40593-s1.doc]

**SNP rs11185644 of RXRA gene is identified for dose-response variability to vitamin D3 supplementation: a randomized clinical trial**

Mingzhi Zhang^1,2,3^,· Lan-Juan Zhao^2,*,+^,· Yu Zhou^2^,· Rhamee Badr^4^, ·Patrice Watson^5^,· An Ye^2^,·Boting Zhou^5^,· Jigang Zhang^2^,· Hong-Wen Deng^2^,· Robert R. Recker^5^,· and Joan M. Lappe^5^

^1^ School of Public Health, Medical College of Soochow University, Department of Epidemiology & Biostatistics, Suzhou, 215123, China.

^2^ Center for Bioinformatics and Genomics, Department of Biostatistics and Bioinformatics, Tulane University, New Orleans, LA, 70112, USA.

^3^ Jiangsu Key Laboratory of Preventive and Translational Medicine for Geriatric Diseases, Medical College of Soochow University, Suzhou, 215123, China.

^4^ School of Medicine, Tulane University, New Orleans, LA, 70112, USA.

^5^ Osteoporosis Research Center, Creighton University, Omaha, NE, 68131, USA.

*** To Whom Correspondence should be addressed:**

Lan-Juan Zhao, PhD, Center for Bioinformatics and Genomics, Department of Biostatistics and Bioinformatics, Tulane University, 1440 Canal Street, Suite 2001, New Orleans, LA 70112, Email:lzhao2@tulane.edu, Phone: 504-988-0252

^+^**The first two authors contributed equally**

**Supplementary Table 1** Identifications for 291 selected SNPs of 15 candidate genes

| Gene | Chromosome | SNP | MAF | Position | Polymorphism | Function |
| --- | --- | --- | --- | --- | --- | --- |
| CYP27A1 | 2 | rs13382651 | 0.417 | 219628190 | A/G | 5'near gene |
| CYP27A1 | 2 | rs6723334 | 0.381 | 219629303 | C/T | 5'near gene |
| CYP27A1 | 2 | rs6751527 | 0.314 | 219630965 | C/G | 5'near gene |
| CYP27A1 | 2 | rs6436084 | 0.383 | 219639782 | A/G | 5'near gene |
| CYP27A1 | 2 | rs7603709 | 0.383 | 219642601 | A/G | 5'near gene |
| CYP27A1 | 2 | rs4674338 | 0.350 | 219647486 | A/G | intron |
| CYP27A1 | 2 | rs933994 | 0.392 | 219650615 | C/T | intron |
| CYP27A1 | 2 | rs17470271 | 0.322 | 219651994 | A/T | intron |
| CYP27A1 | 2 | rs7594289 | 0.350 | 219652285 | A/G | intron |
| CYP27A1 | 2 | rs7568196 | 0.350 | 219652372 | A/G | intron |
| CYP27A1 | 2 | rs12987009 | 0.339 | 219658169 | A/T | intron |
| CYP27A1 | 2 | rs7566656 | 0.430 | 219664518 | A/G | intron |
| CYP27A1 | 2 | rs4646535 | 0.412 | 219664662 | C/T | intron |
| CYP27A1 | 2 | rs12694443 | 0.429 | 219664976 | G/T | intron |
| CYP27A1 | 2 | rs12623740 | 0.407 | 219665714 | A/T | intron |
| CYP27A1 | 2 | rs13013510 | 0.409 | 219667226 | A/G | intron |
| CYP27A1 | 2 | rs12990447 | 0.417 | 219667412 | C/T | intron |
| CYP27A1 | 2 | rs4674344 | 0.433 | 219669874 | A/T | intron |
| CYP27A1 | 2 | rs4674345 | 0.417 | 219671597 | A/G | intron |
| CYP27A1 | 2 | rs6709815 | 0.438 | 219675066 | G/T | intron |
| CYP27A1 | 2 | rs647952 | 0.060 | 219681601 | C/T | 3'near gene |
| CYP27A1 | 2 | rs645163 | 0.150 | 219682257 | A/G | 3'near gene |
| CASR | 3 | rs7648041 | 0.267 | 121899848 | C/T | 5'near gene |
| CASR | 3 | rs4678013 | 0.092 | 121900462 | G/T | 5'near gene |
| CASR | 3 | rs6776158 | 0.288 | 121901849 | A/G | 5'near gene |
| CASR | 3 | rs7628990 | 0.155 | 121904210 | G/T | intron |
| CASR | 3 | rs1501898 | 0.283 | 121905362 | A/C | intron |
| CASR | 3 | rs6438705 | 0.181 | 121906652 | A/G | intron |
| CASR | 3 | rs1847029 | 0.305 | 121907493 | A/G | intron |
| CASR | 3 | rs4678029 | 0.248 | 121908433 | C/T | intron |
| CASR | 3 | rs4678031 | 0.195 | 121909290 | C/T | intron |
| CASR | 3 | rs17282008 | 0.232 | 121909829 | C/G | intron |
| CASR | 3 | rs1501900 | 0.119 | 121910627 | A/T | intron |
| CASR | 3 | rs1973490 | 0.402 | 121910936 | A/T | intron |
| CASR | 3 | rs4678035 | 0.182 | 121911069 | A/C | intron |
| CASR | 3 | rs9826770 | 0.052 | 121913114 | C/T | intron |
| CASR | 3 | rs6764205 | 0.682 | 121914666 | C/T | intron |
| CASR | 3 | rs6438706 | 0.069 | 121916108 | A/G | intron |
| CASR | 3 | rs7617603 | 0.050 | 121916543 | C/T | intron |
| CASR | 3 | rs1814740 | 0.487 | 121918490 | A/G | intron |
| CASR | 3 | rs16832787 | 0.252 | 121919739 | A/G | intron |
| CASR | 3 | rs7614486 | 0.242 | 121924383 | G/T | intron |
| CASR | 3 | rs17203502 | 0.433 | 121927744 | A/G | intron |
| CASR | 3 | rs17203516 | 0.190 | 121932456 | A/G | intron |
| CASR | 3 | rs6438712 | 0.242 | 121933504 | A/G | intron |
| CASR | 3 | rs9820206 | 0.241 | 121933832 | A/G | intron |
| CASR | 3 | rs6799828 | 0.432 | 121935407 | G/T | intron |
| CASR | 3 | rs3845918 | 0.296 | 121935845 | A/G | intron |
| CASR | 3 | rs13327652 | 0.301 | 121936369 | A/G | intron |
| CASR | 3 | rs1501892 | 0.239 | 121938902 | C/T | intron |
| CASR | 3 | rs9866419 | 0.283 | 121940208 | A/G | intron |
| CASR | 3 | rs9875636 | 0.242 | 121947300 | A/G | intron |
| CASR | 3 | rs6764544 | 0.288 | 121948113 | A/G | intron |
| CASR | 3 | rs6768109 | 0.275 | 121949400 | C/T | intron |
| CASR | 3 | rs7639847 | 0.239 | 121949969 | A/G | intron |
| CASR | 3 | rs4677900 | 0.195 | 121953793 | C/T | intron |
| CASR | 3 | rs1354162 | 0.133 | 121954076 | A/C | intron |
| CASR | 3 | rs2134223 | 0.111 | 121956952 | C/T | intron |
| CASR | 3 | rs1979869 | 0.305 | 121963284 | A/G | intron |
| CASR | 3 | rs7635128 | 0.095 | 121966232 | C/G | intron |
| CASR | 3 | rs9875101 | 0.299 | 121968089 | C/T | intron |
| CASR | 3 | rs2173961 | 0.305 | 121968846 | G/T | intron |
| CASR | 3 | rs7644981 | 0.114 | 121969711 | A/G | intron |
| CASR | 3 | rs937626 | 0.308 | 121971801 | A/G | intron |
| CASR | 3 | rs3749203 | 0.5 | 121976493 | C/T | intron |
| CASR | 3 | rs10222633 | 0.473 | 121976925 | A/G | intron |
| CASR | 3 | rs10934578 | 0.296 | 121977281 | G/T | intron |
| CASR | 3 | rs3804592 | 0.137 | 121979228 | A/G | intron |
| CASR | 3 | rs12485716 | 0.308 | 121979689 | A/G | intron |
| CASR | 3 | rs3749207 | 0.336 | 121980241 | C/T | intron |
| CASR | 3 | rs2036399 | 0.150 | 121983606 | C/T | intron |
| CASR | 3 | rs3804593 | 0.169 | 121984020 | A/G | intron |
| CASR | 3 | rs11716910 | 0.372 | 121987618 | A/G | intron |
| CASR | 3 | rs1393198 | 0.097 | 121987931 | C/T | intron |
| CASR | 3 | rs13324814 | 0.150 | 121988119 | A/G | intron |
| CASR | 3 | rs2202127 | 0.327 | 121988354 | A/G | intron |
| CASR | 3 | rs4678173 | 0.371 | 121991138 | A/C | intron |
| CASR | 3 | rs3792288 | 0.150 | 121993431 | A/G | intron |
| CASR | 3 | rs11715859 | 0.114 | 121993836 | A/G | intron |
| CASR | 3 | rs2279802 | 0.076 | 121994940 | A/G | intron |
| CASR | 3 | rs13093602 | 0.068 | 121995414 | C/T | intron |
| CASR | 3 | rs2221266 | 0.349 | 121996815 | C/T | intron |
| CASR | 3 | rs3792291 | 0.067 | 121997267 | C/T | intron |
| CASR | 3 | rs3804595 | 0.142 | 121999650 | C/G | intron |
| CASR | 3 | rs7647405 | 0.049 | 122000240 | C/T | intron |
| CASR | 3 | rs4678174 | 0.327 | 122000870 | C/T | intron |
| CASR | 3 | rs2270916 | 0.150 | 122001098 | C/T | intron |
| CASR | 3 | rs2134224 | 0.137 | 122002177 | C/T | intron |
| CASR | 3 | rs1801725 | 0.133 | 122003756 | T/G | missense |
| CASR | 3 | rs9740 | 0.363 | 122005052 | A/G | intron |
| GC | 4 | rs4694105 | 0.317 | 72592214 | C/T | 3'near gene |
| GC | 4 | rs13117483 | 0.250 | 72594801 | C/T | 3'near gene |
| GC | 4 | rs12512631 | 0.396 | 72601331 | C/T | 3'near gene |
| GC | 4 | rs1491711 | 0.367 | 72602252 | C/G | 3'near gene |
| GC | 4 | rs222049 | 0.104 | 72605955 | C/G | 3'near gene |
| GC | 4 | rs2070741 | 0.093 | 72607678 | A/C | intron |
| GC | 4 | rs705117 | 0.133 | 72608114 | A/G | intron |
| GC | 4 | rs12640179 | 0.100 | 72612686 | C/G | intron |
| GC | 4 | rs12644050 | 0.062 | 72612910 | C/T | intron |
| GC | 4 | rs705119 | 0.417 | 72613035 | A/C | intron |
| GC | 4 | rs1491709 | 0.092 | 72613565 | C/T | intron |
| GC | 4 | rs705120 | 0.417 | 72614139 | G/T | intron |
| GC | 4 | rs2298850 | 0.271 | 72614266 | C/G | intron |
| GC | 4 | rs222040 | 0.422 | 72616931 | C/T | intron |
| GC | 4 | rs4588 | 0.292 | 72618323 | A/C | missense |
| GC | 4 | rs17830803 | 0.084 | 72618984 | C/G | intron |
| GC | 4 | rs16846943 | 0.109 | 72619995 | A/C | intron |
| GC | 4 | rs222035 | 0.442 | 72621673 | A/C | intron |
| GC | 4 | rs222003 | 0.104 | 72626087 | C/G | intron |
| GC | 4 | rs2276461 | 0.083 | 72628968 | C/T | intron |
| GC | 4 | rs705124 | 0.123 | 72632642 | C/T | intron |
| GC | 4 | rs222014 | 0.108 | 72632930 | A/G | intron |
| GC | 4 | rs222016 | 0.125 | 72634975 | A/G | intron |
| GC | 4 | rs222020 | 0.167 | 72636271 | C/T | intron |
| GC | 4 | rs16847015 | 0.050 | 72638824 | A/C | intron |
| GC | 4 | rs1352843 | 0.112 | 72642954 | C/T | intron |
| GC | 4 | rs1155563 | 0.325 | 72643487 | C/T | intron |
| GC | 4 | rs222029 | 0.175 | 72644961 | A/G | intron |
| GC | 4 | rs2298849 | 0.212 | 72648851 | C/T | intron |
| GC | 4 | rs3733359 | 0.067 | 72649773 | C/T | 5'near gene |
| GC | 4 | rs16847028 | 0.136 | 72651525 | A/G | 5'near gene |
| GC | 4 | rs16847050 | 0.133 | 72667331 | C/T | 5'near gene |
| TNF | 6 | rs1799964 | 0.210 | 31542308 | C/T | 5'near gene |
| TNF | 6 | rs1800629 | 0.220 | 31543031 | A/G | 5'near gene |
| TNF | 6 | rs3093662 | 0.070 | 31544189 | A/G | intron |
| CYP3A4 | 7 | rs3735451 | 0.446 | 99355974 | A/G | intron |
| CYP3A4 | 7 | rs2242480 | 0.083 | 99361465 | C/T | intron |
| CYP3A4 | 7 | rs4646437 | 0.133 | 99365082 | C/T | intron |
| CYP3A4 | 7 | rs2687116 | 0.053 | 99365942 | G/T | intron |
| CYP3A4 | 7 | rs35599367 | 0.057 | 99366315 | C/T | intron |
| CYP3A4 | 7 | rs2740574 | 0.109 | 99382095 | A/G | 5'near gene |
| RXRA | 9 | rs10785870 | 0.237 | 137205113 | C/T | 5'near gene |
| RXRA | 9 | rs11185644 | 0.108 | 137210863 | C/T | 5'near gene |
| RXRA | 9 | rs4917347 | 0.225 | 137214071 | C/G | 5'near gene |
| RXRA | 9 | rs11185647 | 0.208 | 137215828 | A/G | 5'near gene |
| RXRA | 9 | rs872298 | 0.225 | 137222136 | A/G | intron |
| RXRA | 9 | rs3818738 | 0.225 | 137225728 | C/T | intron |
| RXRA | 9 | rs3818739 | 0.225 | 137225971 | C/T | intron |
| RXRA | 9 | rs3818740 | 0.225 | 137226093 | C/T | intron |
| RXRA | 9 | rs10881577 | 0.223 | 137227588 | C/T | intron |
| RXRA | 9 | rs881657 | 0.225 | 137230016 | C/T | intron |
| RXRA | 9 | rs4917352 | 0.225 | 137232029 | C/T | intron |
| RXRA | 9 | rs10881578 | 0.225 | 137232535 | A/G | intron |
| RXRA | 9 | rs10881580 | 0.220 | 137234670 | A/G | intron |
| RXRA | 9 | rs4917353 | 0.225 | 137237294 | A/T | intron |
| RXRA | 9 | rs4917354 | 0.225 | 137237660 | C/T | intron |
| RXRA | 9 | rs11185659 | 0.117 | 137243382 | C/T | intron |
| RXRA | 9 | rs7864987 | 0.142 | 137248165 | C/T | intron |
| RXRA | 9 | rs7871655 | 0.142 | 137252786 | C/G | intron |
| RXRA | 9 | rs10881582 | 0.169 | 137256078 | A/G | intron |
| RXRA | 9 | rs10881583 | 0.183 | 137267438 | C/T | intron |
| RXRA | 9 | rs11102986 | 0.158 | 137285503 | A/G | intron |
| RXRA | 9 | rs11103473 | 0.333 | 137287628 | A/T | intron |
| RXRA | 9 | rs3132300 | 0.178 | 137295484 | C/T | intron |
| RXRA | 9 | rs3132299 | 0.158 | 137298814 | C/G | intron |
| RXRA | 9 | rs1805352 | 0.271 | 137299948 | A/C | intron |
| RXRA | 9 | rs3132296 | 0.292 | 137302630 | A/G | intron |
| RXRA | 9 | rs3118540 | 0.227 | 137307105 | C/T | intron |
| RXRA | 9 | rs3118536 | 0.159 | 137308461 | A/C | intron |
| RXRA | 9 | rs12004786 | 0.071 | 137310235 | A/G | intron |
| RXRA | 9 | rs4240705 | 0.295 | 137311399 | A/G | intron |
| RXRA | 9 | rs34677682 | 0.067 | 137316391 | A/G | intron |
| RXRA | 9 | rs3118571 | 0.333 | 137318177 | A/G | intron |
| RXRA | 9 | rs3118570 | 0.167 | 137320854 | A/C | intron |
| RXRA | 9 | rs1536475 | 0.172 | 137321155 | A/G | intron |
| RXRA | 9 | rs35603635 | 0.136 | 137323201 | G/T | intron |
| RXRA | 9 | rs3132294 | 0.208 | 137324119 | A/G | intron |
| RXRA | 9 | rs877954 | 0.325 | 137326407 | A/G | intron |
| RXRA | 9 | rs1805348 | 0.062 | 137328441 | A/G | synonymous |
| RXRA | 9 | rs1045570 | 0.167 | 137332310 | G/T | 3'UTR |
| RXRA | 9 | rs3118523 | 0.208 | 137335521 | A/G | 3'near gene |
| RXRA | 9 | rs3118526 | 0.117 | 137337357 | C/T | 3'near gene |
| RXRB | 9 | rs9277935 | 0.217 | 33160425 | G/T | 3'near gene |
| RXRB | 9 | rs6531 | 0.317 | 33163451 | T/C | synonymous |
| RXRB | 9 | rs3117040 | 0.317 | 33164735 | G/T | intron |
| RXRB | 9 | rs2076310 | 0.225 | 33166034 | C/T | intron |
| CYP2R1 | 11 | rs11023374 | 0.267 | 14903635 | C/T | intron |
| CYP2R1 | 11 | rs7935792 | 0.100 | 14909460 | A/C | intron |
| CYP2R1 | 11 | rs10500804 | 0.458 | 14910272 | G/T | intron |
| CYP2R1 | 11 | rs7129781 | 0.103 | 14912416 | C/T | intron |
| CYP2R1 | 11 | rs10741657 | 0.373 | 14914877 | A/G | 5'near gene |
| CYP2R1 | 11 | rs2060793 | 0.367 | 14915309 | A/G | 5'near gene |
| CYP2R1 | 11 | rs11819875 | 0.223 | 14917297 | G/T | 5'near gene |
| CYP2R1 | 11 | rs16930625 | 0.125 | 14918308 | A/G | 5'near gene |
| CYP2R1 | 11 | rs10766197 | 0.465 | 14921880 | A/G | 5'near gene |
| CYP2R1 | 11 | rs7125348 | 0.129 | 14924665 | A/C | 5'near gene |
| CYP2R1 | 11 | rs952301 | 0.408 | 14928267 | C/G | 5'near gene |
| CYP2R1 | 11 | rs11023380 | 0.438 | 14930058 | A/G | 5'near gene |
| PTH | 11 | rs6256 | 0.125 | 13514053 | A/C | synonymous |
| PTH | 11 | rs6254 | 0.336 | 13514263 | A/G | intron |
| PTH | 11 | rs694 | 0.354 | 13514417 | A/G | intron |
| PTH | 11 | rs177706 | 0.342 | 13514505 | A/G | intron |
| PTH | 11 | rs192802 | 0.336 | 13516194 | C/T | intron |
| PTH | 11 | rs751610 | 0.196 | 13516379 | A/G | intron |
| PTH | 11 | rs2593570 | 0.347 | 13518591 | A/C | 5'near gene |
| FGF23 | 12 | rs13312810 | 0.050 | 4476169 | C/G | 3'down stream |
| FGF23 | 12 | rs13312770 | 0.180 | 4485251 | A/G | intron |
| FGF23 | 12 | rs17700494 | 0.066 | 4487391 | G/T | intron |
| FGF23 | 12 | rs12812339 | 0.300 | 4490845 | A/C | 5'near gene |
| VDR | 12 | rs1859281 | 0.110 | 48215749 | C/T | 3'near gene |
| VDR | 12 | rs2544027 | 0.460 | 48216529 | C/T | 3'near gene |
| VDR | 12 | rs11540149 | 0.070 | 48236663 | A/G | 3'UTR |
| VDR | 12 | rs3847987 | 0.133 | 48238068 | A/C | 3'UTR |
| VDR | 12 | rs11574113 | 0.130 | 48238899 | C/G | intron |
| VDR | 12 | rs1544410 | 0.442 | 48239834 | A/G | intron |
| VDR | 12 | rs12721365 | 0.058 | 48240232 | T/C | synonymous |
| VDR | 12 | rs2525044 | 0.495 | 48242255 | C/T | intron |
| VDR | 12 | rs7963776 | 0.415 | 48243376 | A/G | intron |
| VDR | 12 | rs7967152 | 0.425 | 48244183 | A/C | intron |
| VDR | 12 | rs7971418 | 0.425 | 48245234 | A/C | intron |
| VDR | 12 | rs7305032 | 0.497 | 48249860 | A/G | intron |
| VDR | 12 | rs11168268 | 0.400 | 48251811 | C/T | intron |
| VDR | 12 | rs2248098 | 0.433 | 48253355 | C/T | intron |
| VDR | 12 | rs12370156 | 0.471 | 48254132 | C/T | intron |
| VDR | 12 | rs2239182 | 0.483 | 48255410 | A/G | intron |
| VDR | 12 | rs2107301 | 0.283 | 48255569 | C/T | intron |
| VDR | 12 | rs2239181 | 0.108 | 48255948 | G/T | intron |
| VDR | 12 | rs2239181 | 0.108 | 48256045 | C/G | intron |
| VDR | 12 | rs2239179 | 0.408 | 48257765 | A/G | intron |
| VDR | 12 | rs12717991 | 0.408 | 48259125 | A/G | intron |
| VDR | 12 | rs886441 | 0.192 | 48262963 | C/T | intron |
| VDR | 12 | rs2238138 | 0.270 | 48264492 | C/T | intron |
| VDR | 12 | rs3819545 | 0.500 | 48265005 | C/T | intron |
| VDR | 12 | rs3782905 | 0.329 | 48266167 | C/G | intron |
| VDR | 12 | rs2239186 | 0.385 | 48269409 | C/T | intron |
| VDR | 12 | rs10875693 | 0.317 | 48269649 | A/T | intron |
| VDR | 12 | rs6580642 | 0.167 | 48270595 | C/T | intron |
| VDR | 12 | rs2408876 | 0.325 | 48273564 | C/T | intron |
| VDR | 12 | rs2254210 | 0.454 | 48273713 | A/G | intron |
| VDR | 12 | rs11574044 | 0.125 | 48275833 | G/T | intron |
| VDR | 12 | rs11574042 | 0.143 | 48276178 | C/G | intron |
| VDR | 12 | rs2238136 | 0.233 | 48277712 | A/G | intron |
| VDR | 12 | rs2238135 | 0.280 | 48278189 | C/G | intron |
| VDR | 12 | rs2853564 | 0.425 | 48278486 | C/T | intron |
| VDR | 12 | rs4760648 | 0.498 | 48280664 | C/T | intron |
| VDR | 12 | rs11168287 | 0.495 | 48285413 | A/G | intron |
| VDR | 12 | rs7302235 | 0.477 | 48292837 | C/T | intron |
| VDR | 12 | rs10875695 | 0.240 | 48293036 | A/C | intron |
| VDR | 12 | rs11168292 | 0.347 | 48293604 | C/G | intron |
| VDR | 12 | rs4760655 | 0.333 | 48294130 | A/G | intron |
| VDR | 12 | rs17882106 | 0.100 | 48294412 | C/T | intron |
| VDR | 12 | rs10783219 | 0.322 | 48295487 | A/T | intron |
| VDR | 12 | rs10083198 | 0.270 | 48295964 | C/T | intron |
| VDR | 12 | rs4760658 | 0.367 | 48296485 | A/G | intron |
| VDR | 12 | rs4516035 | 0.438 | 48299825 | C/T | 5'near gene |
| VDR | 12 | rs7976091 | 0.290 | 48304552 | C/T | 5'near gene |
| VDR | 12 | rs10875702 | 0.167 | 48313018 | A/T | 5'near gene |
| VDR | 12 | rs7311030 | 0.178 | 48317832 | C/T | 5'near gene |
| CYP27B1 | 12 | rs4646537 | 0.052 | 58157280 | A/C | intron |
| CYP27B1 | 12 | rs4646536 | 0.367 | 58157987 | C/T | intron |
| CYP27B1 | 12 | rs8176345 | 0.050 | 58158558 | A/G | synonymous |
| CYP27B1 | 12 | rs703842 | 0.375 | 58162738 | C/T | 5'near gene |
| CYP11A1 | 15 | rs2279357 | 0.268 | 74630622 | C/T | intron |
| CYP11A1 | 15 | rs12917295 | 0.434 | 74632283 | C/G | intron |
| CYP11A1 | 15 | rs11632698 | 0.367 | 74637866 | A/G | intron |
| CYP11A1 | 15 | rs12916123 | 0.270 | 74638964 | A/C | intron |
| CYP11A1 | 15 | rs1484215 | 0.071 | 74640108 | C/T | intron |
| CYP11A1 | 15 | rs6495096 | 0.342 | 74640646 | C/G | intron |
| CYP11A1 | 15 | rs4887139 | 0.127 | 74661944 | A/G | 5'near gene |
| CYP1A1 | 15 | rs4646421 | 0.080 | 74723851 | C/T | intron |
| CYP1A1 | 15 | rs1048943 | 0.067 | 75012984 | C/G | missense |
| CYP1A1 | 15 | rs2606345 | 0.363 | 75017175 | A/C | intron |
| CYP24A1 | 20 | rs1870969 | 0.483 | 52754457 | A/T | 3'near gene |
| CYP24A1 | 20 | rs6097797 | 0.104 | 52763331 | C/T | 3'near gene |
| CYP24A1 | 20 | rs6097801 | 0.104 | 52767434 | A/G | 3'near gene |
| CYP24A1 | 20 | rs6097805 | 0.500 | 52768496 | A/G | 3'near gene |
| CYP24A1 | 20 | rs4809957 | 0.158 | 52771170 | A/G | 3'UTR |
| CYP24A1 | 20 | rs927650 | 0.433 | 52772740 | A/G | intron |
| CYP24A1 | 20 | rs1570669 | 0.263 | 52774426 | A/G | intron |
| CYP24A1 | 20 | rs6068812 | 0.006 | 52774634 | C/T | missense |
| CYP24A1 | 20 | rs2296239 | 0.454 | 52775527 | A/G | synonymous |
| CYP24A1 | 20 | rs751090 | 0.146 | 52776738 | A/G | intron |
| CYP24A1 | 20 | rs2585439 | 0.236 | 52777118 | A/G | intron |
| CYP24A1 | 20 | rs2209314 | 0.267 | 52778960 | C/T | intron |
| CYP24A1 | 20 | rs3886163 | 0.129 | 52780192 | C/T | intron |
| CYP24A1 | 20 | rs6068816 | 0.337 | 52781090 | A/G | synonymous |
| CYP24A1 | 20 | rs4809958 | 0.136 | 52782437 | G/T | intron |
| CYP24A1 | 20 | rs2762941 | 0.492 | 52783651 | A/G | intron |
| CYP24A1 | 20 | rs2181874 | 0.237 | 52784477 | A/G | intron |
| CYP24A1 | 20 | rs4809959 | 0.475 | 52785858 | A/G | intron |
| CYP24A1 | 20 | rs4809960 | 0.225 | 52786072 | C/T | intron |
| CYP24A1 | 20 | rs2296241 | 0.497 | 52786218 | T/C | synonymous |
| CYP24A1 | 20 | rs6022999 | 0.233 | 52788012 | A/G | intron |
| CYP24A1 | 20 | rs35051736 | 0.020 | 52788078 | A/G | missense |
| CYP24A1 | 20 | rs2248359 | 0.375 | 52791518 | C/T | 5'near gene |
| CYP24A1 | 20 | rs2426498 | 0.133 | 52796681 | C/G | 5'near gene |
| CYP24A1 | 20 | rs2021940 | 0.417 | 52798744 | C/T | 5'near gene |
| CYP24A1 | 20 | rs6023005 | 0.339 | 52800866 | A/G | 5'near gene |
| CYP24A1 | 20 | rs765059 | 0.250 | 52804511 | C/G | 5'near gene |
| CYP24A1 | 20 | rs6023012 | 0.432 | 52806573 | A/G | 5'near gene |
| CYP24A1 | 20 | rs1555439 | 0.186 | 52809296 | G/T | 5'near gene |

The information of identification comes from Genome Build Version 36.2.
